# First impressions of the foundation interim year 1 postings: positives, pitfalls, and perils

**DOI:** 10.1080/10872981.2020.1785116

**Published:** 2020-06-25

**Authors:** Sofian Youssef, Syed Zaidi, Sandesh Shrestha, Christy Varghese, Sriram Rajagopalan

**Affiliations:** a Keele University School of Medicine, University Hospitals of North Midlands NHS Trust, Stoke-on-Trent UK, England; b Keele University School of Medicine, Shrewsbury and Telford Hospital NHS Trust, Shrewsbury, UK, England

**Keywords:** COVID-19, FiY1, junior doctor, foundation training, redeployment

## Abstract

COVID-19 has placed an increased burden on the NHS. Changes were made to expand patient capacity including hospital restructuring, cancellation of most elective surgeries and early graduation of final year medical students.**^1^** The UK foundation programme (UKFP) curated a new training position for graduates as foundation interim year 1 (FiY1) doctors, where they voluntarily work in paid positions prior to entering formal foundation year 1 (FY1) roles.**^2^** Expediting the process of fulfilling these positions, the General Medical Council facilitated early provisional registration of doctors. We discuss the positives, pitfalls, and perils of the new roles and the first impressions of three newly qualified FiY1 s in medical, obstetrics and gynaecology and surgical posts, a surgical FY1 doctor and a clinical supervisor in surgery.

## Medical FiY1

Within general internal medicine (gastroenterology), a typical day starts with a ward round at 9am, where an FiY1 would be expected to participate at the same level of FY1 s, preparing and presenting patient notes to the consultant. Jobs carried out by an FiY1 on this ward include ordering investigations, requesting specialist reviews, writing discharge letters, and carrying out procedures such as venesection, cannulation, and sometimes ascitic taps and drains under appropriate supervision.

## Obstetrics and Gynaecology FiY1

FiY1’s roles in Obstetrics and Gynaecology (O&G) depend on the subspecialty. Overall a usual day will start at 8 am and finish by 4 pm. In obstetrics, the care of patients is mostly led by registrars, so FiY1 s are largely supernumerary. Following labour ward round, FiY1 s either assist in the caesarean section list or attend antenatal and postnatal wards to help the registrar review patients for discharge. Gynaecology runs in a similar fashion, with greater emphasis on clerking patients and executing management plans implemented by seniors.

## Surgical FiY1

Surgical jobs have been transformed most by the COVID-19 pandemic; Trauma & Orthopaedics (T&O) is among the wards restructured to facilitate COVID-19 patients, having cancelled most elective procedures[1]. Due to these changes, there are far fewer patients requiring ongoing ward care and thus reduced administrative responsibilities. Moreover, a typical day for an FiY1 in the T&O ward begins at 10 am and ends at 6 pm and involves clerking patients, discussing management plans with seniors, prescribing and making relevant referrals. Educational support has also been provided through regular departmental teaching.

## Surgical FY1

From the perspective of a surgical FY1 doctor working on a ward with an FiY1, the role of an interim F1 is much the same to that of a final year medical student with additional benefits of being able to prescribe, request investigations and complete medical documentation. This has radically reduced the workload for junior doctors and the department as a whole.

## Clinical supervisor

From a clinician’s standpoint, the FiY1 post was a welcome initiative, notably to fulfil the roles of doctors deployed to other wards and those absent due to sickness or self-isolation. As an educator, the trainer-trainee pathways for this post were not clearly established due to the accelerated process of its creation. It appears contradictory for a training position like this to not require a formally arranged meeting with a supervisor, although it has remained the main priority to provide a sustainable and safe service, which holds greater precedence beyond the roles played in educational and supervisory positions.

## Positives

In line with established foundation training support systems, FiY1 doctors were granted access to SCRIPT, an eLearning training programme. In addition, all graduates were allocated clinical supervisors for personal guidance. The new doctors have also been provided with early access to Horus e-portfolio, an electronic log of FY1 competencies needed for progression to full GMC registration[2].

Undoubtedly, the roles have provided opportunities to gain extra clinical experience. Graduates often require time to accustom to new responsibilities; FiY1 encompasses this phase of adjustment. The new role also provides continuity of care that temporarily cannot be provided by junior doctors on emergency COVID-19 rotas. Further benefits include the position being paid and no on-call responsibilities or out of hours work. Additionally, foundation trainees have been exceedingly supportive and trusts also preferentially placed doctors in COVID-19 negative wards as a further protective measure.

## Pitfalls

Whilst the UKFP recommended the development of a ‘buddy’ system, this has proven a difficult concept to implement[2,3]. Foundation doctors work under vastly varied rotas, notably the new emergency COVID-19 rotas, that is incompatible with that of FiY1s. As such, this presents challenges for the new graduates if no immediate buddy help is available.

Offloading excess burden caused by COVID-19 on trusts was the main reason for the creation of the new role, but posts at this trust were commenced after the peak of the virus. Rather, it is more likely that the role has facilitated graduates to accustom themselves to work as junior doctors. The question remains whether the introduction of these new roles has provided a return on investment for trusts, who were at one point struggling with debt [2,3].

## Perils

One of the major concerns surrounding FiY1 is whether there is sufficient justification in exposing the new doctors to COVID-19. Whilst occupational health checks are provided for all doctors, some graduates may have felt obliged to take up the roles despite having conditions increasing risks.

## Summary

Moreover, the weeks of heightened NHS burden have been chaotic. In future, it may be feasible for the NHS to replicate this role on a voluntary or funded basis as a means of enhancing clinical experiences and portfolios, particularly if juniors were allocated to the specialty of their choice. Whilst there are obstacles impeding this such as the usual timing of graduation, the options could still be made available for the future doctors.
